# A new interstitial species of the genus *Caecianiropsis* Menzies & Pettit, 1956 (Isopoda, Asellota) from Korea

**DOI:** 10.3897/zookeys.832.30241

**Published:** 2019-03-19

**Authors:** Jeongho Kim, Wonchoel Lee, Ivana Karanovic

**Affiliations:** 1 Department of Life Science, Hanyang University, 222 Wangsipriro, Seongdonggu, Seoul, South Korea Hanyang University Seoul South Korea; 2 Institute for Marine and Antarctic Studies, University of Tasmania, Hobart, Tasmania, 7001, Australia University of Tasmania Hobart Australia

**Keywords:** East Asia, Janiridae, marine benthos, taxonomic key

## Abstract

A new interstitial species, *Caecianiropsisgoseongensis***sp. n.** is described from littoral off the east coast of Korea (Sea of Japan). The species can be distinguished from its congeners by the number of antennular articles, shape of the male appendix masculina, setation of pereopods, and length ratio of the uropodal rami. To aid species identification a taxonomic key to all species of *Caecianiropsis* Menzies & Pettit, 1956 is also provided as well as a partial 16S mitochondrial ribosome RNA of the new species, which is the first genetic information for the genus.

## Introduction

Menzies and Pettit (1956) established *Caecianiropsis* for the new species *Caecianiropsispsammophila* Menzies & Pettit, 1956 collected from a coarse sand beach in California. This species was distinct from all other members of the family Janiridae by the following morphological characters: an elongated body (6.5 times longer than wide), distolateral extension forming an angular shape, and coiled appendix masculina in the relaxed position. So far only three species were described: *Caecianiropsispsammophila* from northern California (Menzies and Pettit 1956; Kussakin 1988), *C.birsteini* Kussakin, 1979 from Okhotsk Sea (Kussakin 1979, 1988), and *C.ectiformis* (Vanhöffen, 1914) from Kerguelen (Vanhöffen 1914), St. Paul (Kensley 1976), and Marion and Prince Edward Islands (Branch et al. 1991). Wilson and Wägele (1994) provided a brief review of *Caecianiropsis* and divided it into two species groups based on the region from where they were collected. The northern group included *C.psammophila* and *C.birsteini*, and was characterized by a relatively longer antennal flagellum, a similar width of the rostrum and the antennular peduncle, and an angular lateral extension of the male pleopod I. The southern group included only *C.ectiformis*, a species with a compact antennal flagellum, a much broader rostrum, and lacking the lateral extension on the male pleopod. *Caecianiropsisectiformis* was originally described in the genus *Austroniscus* Vanhöffen, 1914 and transferred to its present systematic position by Menzies and Pettit (1956). Nevertheless, Wilson and Wägele (1994) questioned this because of its distinct morphology and suggested a thorough reexamination of the species with the type material.

A new species of *Caecianiropsis* was collected from shallow water of the East Sea (Sea of Japan) near Goseong (Gangwondo, Korea). It has a typical body plan of *Caecianiropsis* but also a unique combination of characters which clearly distinguish the new species from all other congeners. This paper provides an illustrated description of a new species, a revised generic diagnosis, and an identification key to the four species of *Caecianiropsis*. In addition, a partial mitochondrial sequence of 16S ribosome RNA gene was obtained and this may be useful for the future phylogenetic study of *Caecianiropsis*.

## Materials and methods

### Specimen collection and examination

Samples were collected from littoral off the East coast of Korea (depth 15 m), by scuba diving with plastic corer and initially kept in a plastic bag. Sediment was transferred to 250 ml bottles and immediately preserved in 99% ethanol. Sorting from sediment sample and dissection of specimens were done under an Olympus SZX 12 stereo-binocular microscope. Dissected appendages were mounted onto glass slides in lactophenol. The line drawings were prepared using Olympus BX 51compound microscope equipped with a *camera lucida*. All studied material was deposited at the invertebrate collection of the National Institute of Biological Resources (NIBR), Korea. One male and one female were transferred to isoamyl acetate for 20 minutes and dried in a critical-point dryer Hitachi E-1010. Dried specimens were mounted onto a SEM stub and coated with gold using a sputter coater to a thickness of 15–30nm. Coated specimens were examined and photographed with a Hitachi S-3400 scanning electron microscope at Eulji University (Seongnam, Korea). Measurements were made following the method of Riehl and Brandt (2010). All measurements were taken from the dorsal view of line drawings using the distance measurement tools of Adobe Acrobat Professional. The ratios of appendages were given in distal to proximal order, excluding setae. The body ratios were given in anteromedial point to posteromedial point order excluding appendages. Terminology is largely based on Wilson and Wägele (1994). We abbreviated the term ‘unequally bifid’ seta as UB seta.

### DNA extraction and amplification

Two females from the type locality were identified without dissection under Olympus SZX 12 stereomicroscope. Before amplification, specimens were transferred into distilled water for 20 minute to remove ethanol and then minced with a small glass stick. Whole specimens were used to isolate genomic DNA with the aid of the LaboPassTM Kit (COSMO Co. Ltd., Korea) following the manufacturer’s protocols. The 16S rDNA gene was amplified with polymerase chain reaction (PCR) using PCR premix (BIONEER.Co) in TaKaRa PCR thermal cycler (TaKaRa Bio Inc., Otsu, Shiga, Japan). The primers used were 16sar-L (5'‒ CGC CTG TTT AAC AAA AAC AT‒3') and 16sar-H (5'‒CCG GTC TGA ACT CAG ATC ACG T‒3') (Palumbi et al. 1991). The amplification protocol consisted of initial denaturation 94 °C for 2 min, 35 cycles of denaturation 94 °C for 50 sec, annealing at 50 °C for 50 sec, extension at 72 °C for 1 min 20 sec and final extension at 72 °C for 7 min. The final product was stored at 4 °C. Amplifications were confirmed by electrophoresis in 1% agarose gel. The PCR products were purified for sequencing reactions, using the Labopass PCR Purification Kit (COSMO Co. Ltd., Korea) following the guidelines provided with the kit. DNA was sequenced on an ABI automatic capillary sequencer (Macrogen, Seoul, Korea) using the same set of primers.

## Taxonomy

### Suborder Asellota Latreille, 1802

#### Superfamily Janiroidea Sars, 1897

##### Family Janiridae Sars, 1897

###### Genus Caecianiropsis

Taxon classificationAnimaliaIsopodaJaniridae

Menzies & Pettit, 1956

Austroniscus Vanhöffen, 1914: 553; Branch et al. 1991: 28.
Caecianiropsis , Menzies and Pettit 1956: 441; Kensley 1976: 295; Kussakin 1979, 1988: 160; Wilson 1994: 751; Wilson and Wägele 1994: 693.

####### Type species.

*Caecianiropsispsammophila* Menzies & Pettit, 1956

####### Included species.

*C.birsteini* Kussakin, 1979, *C.ectiformis* (Vanhöffen, 1914)

####### Generic diagnosis.

(modified from Wilson and Wägele 1994)

Body six times longer than wide; cephalon with no eye, weakly developed rostrum reaching to middle of antennular article I; pleonite I 0.8 times wider than pereonite VII; pleotelson as wide as pereonite VII; antennula with V‒VII articles, antennal article III with rudimentary scale laterally; mandibular molar process truncate, palp article II medially swollen, with 2‒3 robust setae, median setigerous margin slightly depressed; maxillipedal endite two times longer than wide, distomedially pointed; medial lobes of male pleopod I distally rounded, distolateral edge of hyaline lamella projected; exopod of male pleopod II inserting subdistally on sympod, endopod proximally expanded, appendix masculina more than four times longer than sympod, coiled in relaxed position; endopod of pleopod III with three distal broom setae having distinct gap between medial seta and two lateral setae.

####### Remarks.

Wilson and Wägele (1994) provided a simple note on the morphological affinity between *Caecianiropsis* and *Neojaera* emphasizing a coiled, very much elongate stylet of male pleopod II in male. The major differences between *Neojaera* and *Caecianiropsis* are the presence of distinctly developed uropods showing the elongate sympod, and the much longer endopod in *Caecianiropsis*. In addition, the development of rostrum is also a noticeable difference between the two genera, with *Caecianiropsis* having an elongated rostrum reaching to the middle of the antennular article I, and its width is almost the same as antennula article 1, while *Neojaera* has only a weak anterior protrusion on the rostrum. Other morphological differences between the two genera are as follows: 1) cephalon without visual organ; 2) length of the cephalon as long as its width; 3) all pereonites almost same in length; 4) antenna much longer than antennula (more than twice); 5) lateral margin of the male pleopod I extended, angular form; 6) sympod of the male pleopod II, 2.6 times longer than wide. Wilson and Wägele (1994) also found several similarities between *Caecianiropsis* and *Microjaera* Bocquet & Levi, 1955 including the body form, antennal articulation, and male pleopod II with the elongate and coiled stylet. However, the elongation of the body can be a result of adaptation to the interstitial environment and therefore often evolve convergently. In addition, similar antennal articulation can be found in many other isopod groups. This similarity is also only superficial because *Caecianiropsis* shows a rudimentary scale on article III of antenna, which is lacking in *Microjaera*. On the other hand, elongation of the male stylet is one of the most noticeable characters of *Caecianiropsis* within the family Janiridae. Although, Wilson and Wägele (1994) mentioned that *Microjaeraanisopoda* Bocquet & Levi, 1955 also possesses a similar morphology of male pleopod II, the original description of this species was limited due to the poor illustration of this particular character. Shimomura (2005) described another species, *Microjaeramorii* Shimomura, 2005, but based on non-ovigerous female only; therefore this important male character is missing. Phylogenetic analysis of Janiridae (Wilson 1994) based on 33 morphological characters suggested a close relationship between *Caecianiropsis* and the *Microjaera*. However, this has to be considered with caution because the important male characters are not well described in the latter genus.

###### Caecianiropsis goseongensis sp. n.

Taxon classificationAnimaliaIsopodaJaniridae

http://zoobank.org/948FB812-988F-470C-A52D-11B469BEF7F3

Figures 1 , 2 , 3 , 4 , 5 , 6 , 7 , 8 , 9

####### Type locality.

Shallow water of East Sea (Sea of Japan), Goseong, Gangwondo, Korea, 38°17'43.8"N, 128°33'15.1"E.

####### Material examined.

***Holotype*** : adult male, (NIBRIV0000838292) completely dissected and mounted in lactophenol on eight slides; allotype: non-ovigerous female (NIBRIV0000838293) dissected on three slides; *paratype* 1: adult male (NIBRIV0000838294) dissected on three slides; paratype 2 adult male (NIBRIV0000838295) dissected on two slides; paratype 3: male used for SEM (NIBRIV0000838296).

####### Diagnosis.

Pleotelson 1.45 times longer than wide, 0.23 times of whole body, antennula with seven articles, article VI with one simple seta and two aesthetascs distally, article VII very tiny, with three simple setae and one elongate aesthetasc, male antenna with 29 articles of flagellum, left mandible with five serrate setae in spine low, maxillipedal palp with setal formula as follow: 2:12:7:8:10, pereopod I with setal formula as follow: 5:1:10:13:19:7, pereopod II with setal formula as follow: 8:5:8:11:14:5, distal margin of pleopod I with 38 setae, uropodal exopod 0.5 times of endopod. Female operculum with two setae on medial margin and six setae along distal apex.

####### Description of the male holotype.

**Figure 1.**
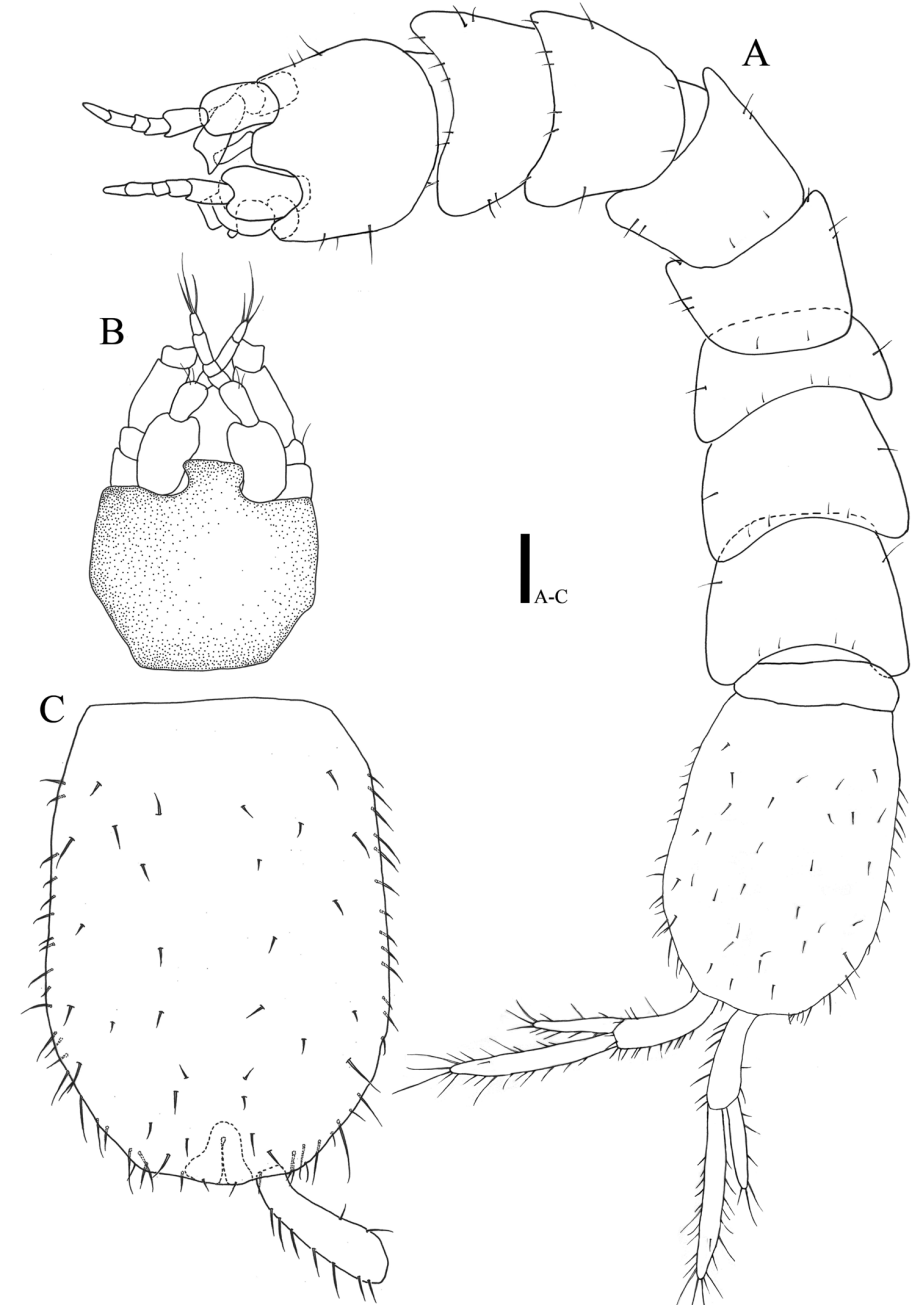
*Caecianiropsisgoseongensis* sp. n. **A** Paratype 1, male, habitus, dorsal, ×200 **B** holotype, male, cephalon, dorsal, ×200 **C** holotype, male, pleotelson, dorsal, ×200, uropod, dorsal, ×600. Scale bar: 100 µm.

*Body* (Fig. 1A): elongate, flattened in dorsal view, color of preserved specimens transparent, total length measured with paratype I 2.05 mm, length six times longer than wide, maximal body width in pereonite V 0.91 times of maximal width of pleotelson, setation of pereonites I‒VII as follows: 10: 10: 10: 8: 6: 6: 6.

*Cephalon* (Fig. 1B): 0.94 times longer than wide and 0.16 times of whole body, anterior margin wider than posterior one; rostrum slightly wider than antennular article I.

*Pleotelson* (Fig. 1C): 1.45 times longer than wide, 0.23 times of whole body, dorsal and lateral margin covered with many setae.

**Figure 2.**
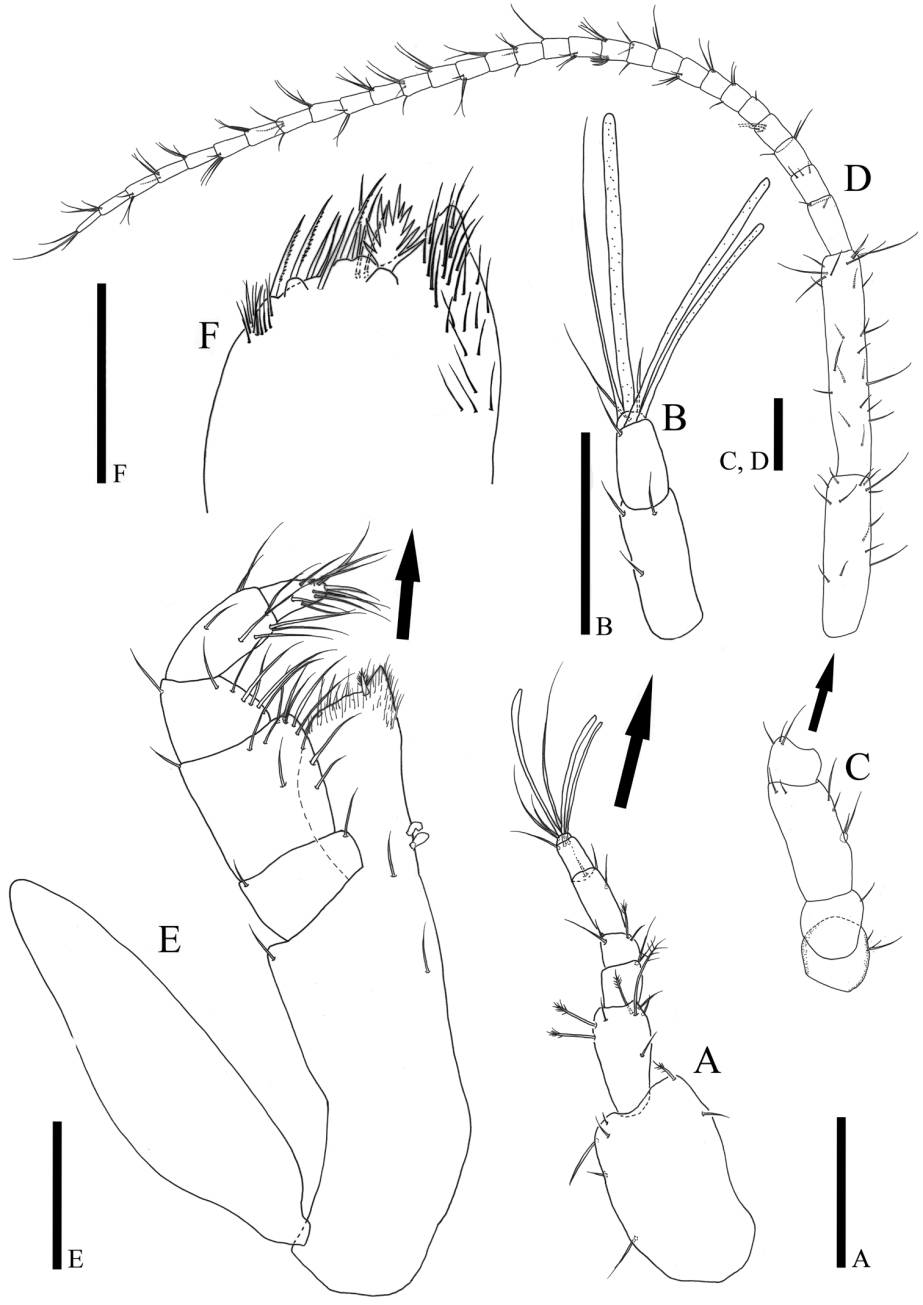
*Caecianiropsisgoseongensis* sp. n. holotype, male. **A** Antennula, dorsal, ×400 **B** antennula articles 5‒7, dorsal, ×600 **C** antennal article 1‒4, dorsal, ×400 **D** antennal article 5, 6 and flagellum, dorsal, ×400 **E** maxilliped, ventral, ×400 **F** paratype 3, male, distal margin of endite, ventral, ×1000. Scale bars: 100 µm (**A–E**), 50 µm (**F**).

*Antennula* (Fig. 2A): seven articles, relative length ratios: 1: 0.97: 0.22: 0.17: 0.36: 0.24: 0.03; article I robust, 1.38 times longer than wide, lateral margin with three simple setae, distal margin with three simple and one broom setae; article II 0.48 times wider than article I 1.89 times longer than wide, with three simple and four broom setae; article III 1.1 times longer than wide, with two simple setae distolaterally; article IV 0.89 times longer than wide, with three simple and one broom seta distally; article V 2.58 times longer than wide, with three simple setae distolaterally; article VI 1.72 times longer than wide, one simple seta and two aesthetascs distally; article VII (Fig. 2B) smallest, with three simple and one elongate aesthetasc on distal end.

*Antenna* (Fig. 2C, D): six peduncle articles and 29 flagellar articles; article I with three mediolateral setae; article II 0.92 longer than wide, with one seta distolaterally; article III twice as long as wide, with four setae distally, rudimentary scale on lateral margin with two setae; article IV 0.71 times longer than wide, with three simple mediolateral setae; article V 3.55 times longer than wide, with 14 simple setae; article VI longest, 5.68 times longer than wide, with 25 simple setae; flagellar article I longest, 2 times longer than wide, with two simple setae distally, setal formula of articles from 2 to 29 as follows: 3: 3: 4: 1: 3: 2: 4: 2: 3: 8: 3: 1: 4: 2: 3: 3: 4: 4: 3: 3: 4: 3: 4: 3: 4: 3: 3: 3.

*Maxilliped* (Fig. 2E): epipodite, narrow, 3.56 times longer than wide, distal end reaching to palp article II; basis 3.8 times longer than wide; endite width 0.83 times of palp article II, two proximomedial coupling hooks, distal margin covered with numerous fine setae, with six simple setae, three serrated setae, and one fan seta (Fig. 2F); palp relative length ratio: 1: 1.77: 1.14: 1.63: 0.91, article I 0.55 times longer than wide, with two simple setae on both distal corners; article II quadrangular, 0.95 times longer than wide, 1.9 times longer than article I, with eleven simple long setae on distomedial margin and one short seta on distolateral corner; article III 1.13 times longer than article I, tapering distally, with six simple long setae along medial margin and one seta on distolateral corner; article IV 2.14 times longer than wide, with eight simple setae on distal margin; article V with eight simple and two robust setae on distal end.

**Figure 3.**
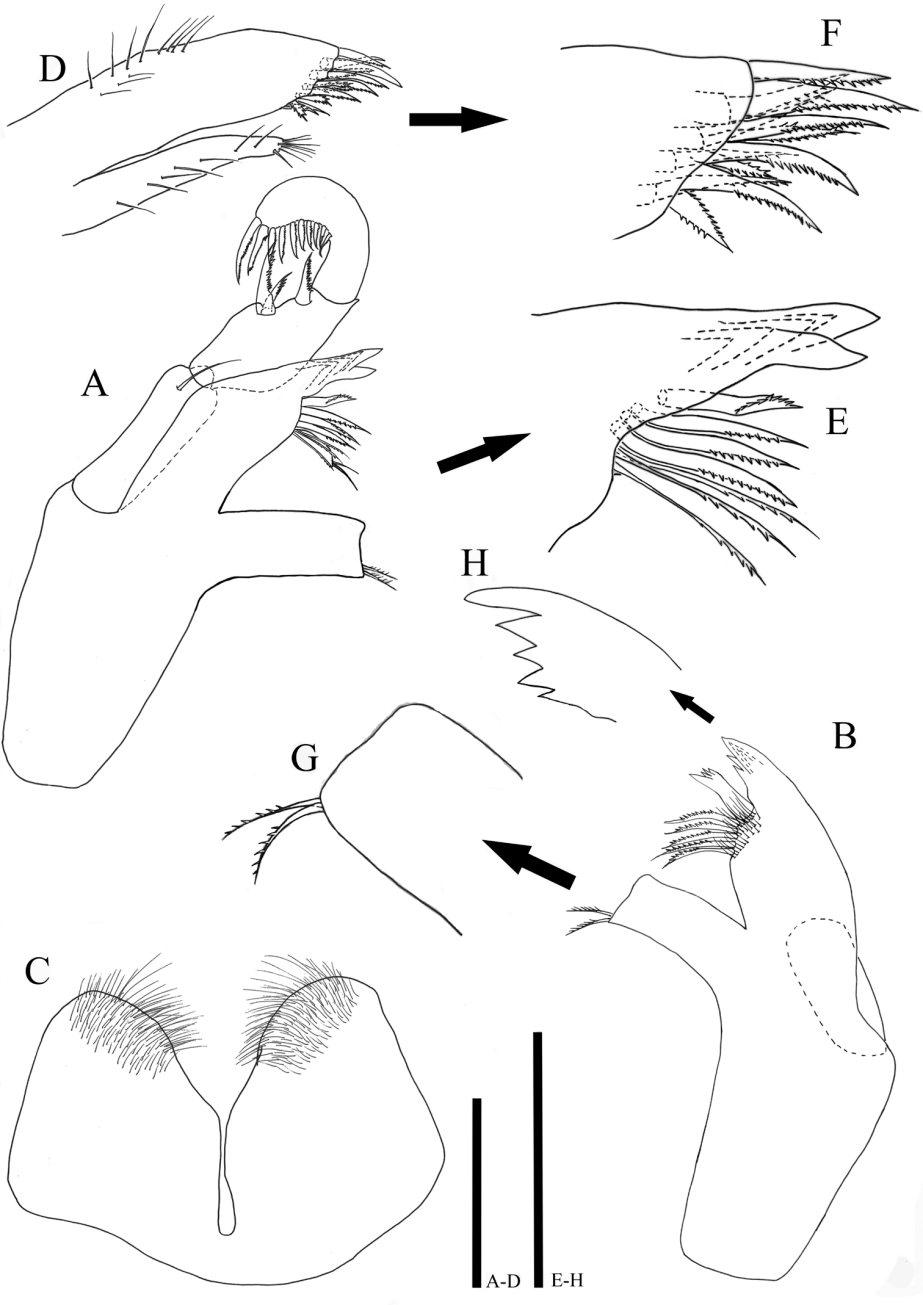
*Caecianiropsisgoseongensis* sp. n. holotype, male. **A** Left mandible, ventral, ×600 **B** right mandible, ventral, ×600 **C** hypopharynx, ventral, ×600 **D** maxillula, ventral, ×600 **E** distal end of maxillula, ventral, ×1000, **F** distal end of left mandible, ventral, ×1000, **G** distal end of *pars molaris*, ventral, ×1000, **H** pars incisiva, lateral, ×1000. Scale bars: 100 µm (**A–D**), 50 µm (**E–H**).

*Mandible* (Fig. 3A, B): body robust, curved inwardly; left mandible (Fig. 3A) pars incisiva with four cusps; lacinia mobilis much smaller than that of right mandible, with three denticulate, robust spines and three serrate setae (Fig. 3E); pars molaris truncate, missing grinding surface, distal tip blunt, with two apical setae; right mandible (Fig. 3B) pars incisiva with five (Fig. 3H), lacinia mobilis smaller than pars incisiva, with five cusps, five serrate setae located below lacinia mobilis, proximal part of setae covered by fine numerous setae, pars molaris with two apical setae (Fig. 3G); palp (Fig. 3A) 0.97 times of body length, inserted on cuticular projection; article I with one seta distally, 3.58 times longer than wide; article II robust, 1.89 times longer than wide, length 0.83 times of article I, maximal width 1.58 times of article I, median margin swollen, with three serrate setae distolaterally, article III laterally curved with ten serrate setae on inner margin.

*Hypopharynx* (Fig. 3C): deep medial incision separating two lobes, much of hairs on distal margin of each lobe.

*Maxillula* (Figs 3D, F): inner endite shorter and more slender than outer one , with one short setae and several hair-like elements on distal apex, along lateral margin with spiny row; outer endite with 12 robust setae on distal margin, most denticulate, some two-sided serrate, along lateral margin with long simple setae.

**Figure 4.**
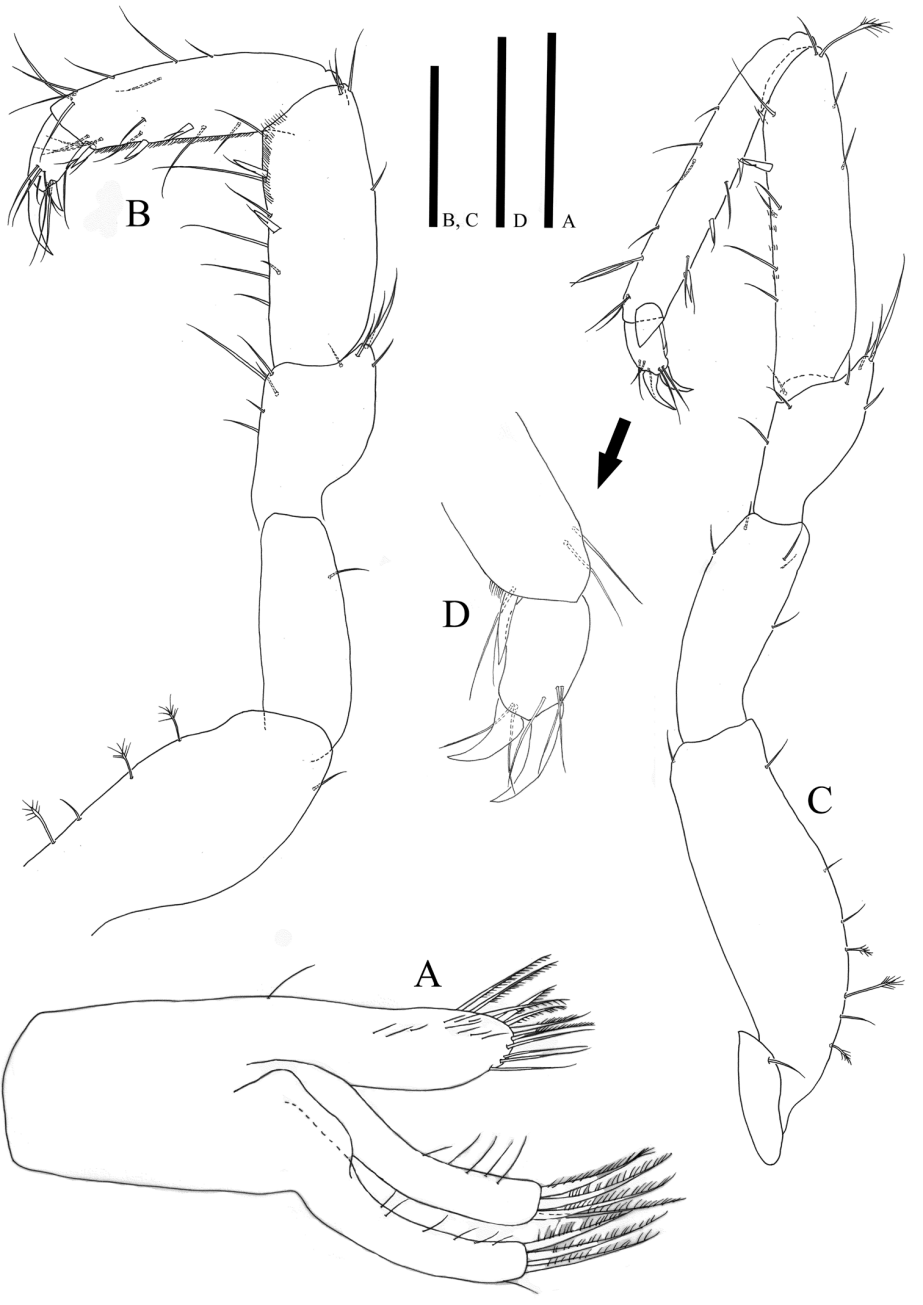
*Caecianiropsisgoseongensis* sp. n. **A** Maxilla, paratype 3, male, ventral, ×600; holotype, male, **B** pereopod 1, dorsal, ×400 **C** pereopod 2, dorsal, ×400 **D** propodus of pereopod 2, ventral, ×1000. Scale bars: 50 µm (**A, D**), 100 µm (**B, C**).

*Maxilla* (Fig. 4A): all rami similar in length, with four serrated setae on distal end of each; mesial ramus coalescent with basis, much thicker than others, with eleven strong setae distally and some of them denticulate; median ramus with four pectinate setae and lateral one with three pectinate setae distally, all rami with fine simple setae along medial margin.

**Figure 5.**
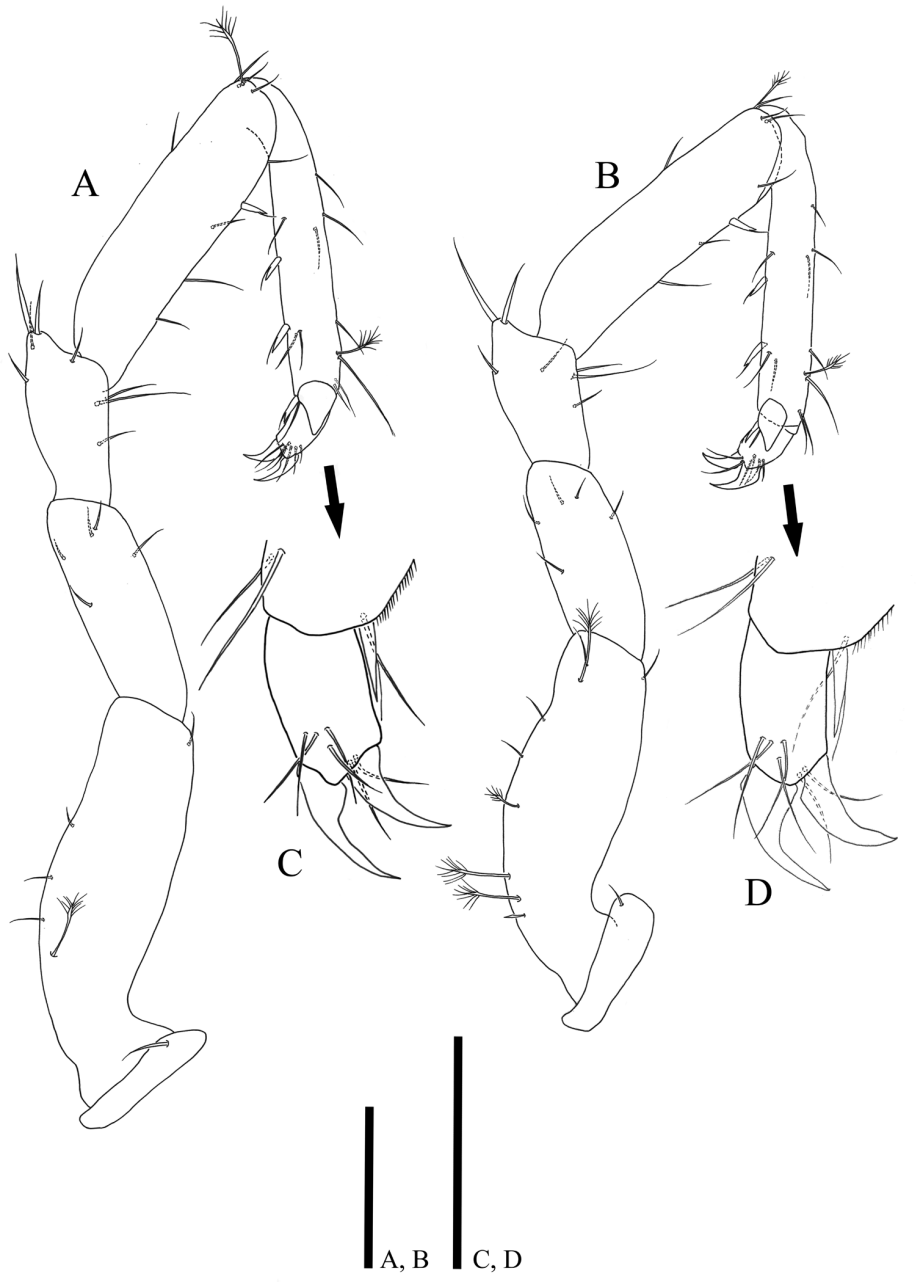
*Caecianiropsisgoseongensis*. holotype, male. **A** Pereopod 3, dorsal, ×400 **B** pereopod 4, dorsal, ×400, **C** propodus of pereopod 3, ventral, ×1000 **D** propodus of pereopod 4, ventral, ×1000. Scale bars: 100 µm (**A, B**), 50µm (**C, D**).

*Anterior pereopods* (Figs 4B, C, 5A, B) inserted on pereon anterolaterally, relative length ratio: 1: 1.12: 1.12: 1.01; width ratio of carpus and propodus: 1: 0.87: 0.87: 0.73/ 1: 0.6: 0.6: 0.55; L/W ratio of articles: basis (2.03: 3.03: 2.97: 2.86), ischium (2.27: 2.57: 2.65: 2.05), merus (1.4: 1.52: 1.75: 1.52), carpus (2.52: 4.21: 3.71: 3.9) propodus (3.21: 6.83: 6.08: 6.17), dactylus (1.5: 1.48: 1.55: 1.45); pereopod I with minute coxa hardly discernable in lateral view (Fig. 9A); coxae of pereopods II‒IV clearly visible in lateral view with small simple seta; dactylus of pereopods II‒IV partly covered by articular plate projected from distal margin of propodus.

*Pereopod I* (Fig. 4B) basis with three broom and two simple setae; ischium with one seta on dorsal margin; merus with four setae of different lengths on distodorsal corner and five setae on ventral margin; carpus dorsal margin with four setae, two UB setae on ventral margin with six simple setae of different lengths, ventral margin with hairs; propodus with eight simple setae along dorsal margin, ventral margin with seven simple and four UB setae; dactylus tapering distally, dorsal margin with four setae of different lengths, ventral side with three setae, two claws on distal apex with different lengths.

*Pereopod II* (Fig. 4C): basis dorsal margin with four simple and three broom setae and with one short seta on distal corner; ischium with five simple setae, merus distal margin with eight setae of different lengths; carpus dorsal margin with three simple and one broom seta, ventral margin with five simple and two UB setae; propodus with eight setae along dorsal margin and two simple and four UB setae along ventral margin; dactylus (Fig. 4D) with two claws and five simple setae.

*Pereopod III* (Fig. 5A): basis dorsal margin with three simple and one broom setae, and with one short seta on distal corner; ischium with five simple setae; merus distal margin with eight setae of different lengths; carpus dorsal margin with one broom and three simple setae, ventral margin with one UB and four simple setae; propodus dorsal margin with seven simple and one broom setae, four simple and three UB setae along ventral margin; dactylus (Fig. 5C) with two claws and six simple setae.

*Pereopod IV* (Fig. 5B): basis with three simple and five broom setae on dorsal margin, one short seta on distal corner; ischium with five simple setae on distal margin; merus with seven setae of different length on distal margin; carpus dorsal margin with four simple and one broom seta, ventral margin with three simple and one UB seta; propodus dorsal margin with seven simple and one broom seta, four simple and three UB setae along ventral margin; dactylus (Fig. 5D) with two claws and six simple setae.

**Figure 6.**
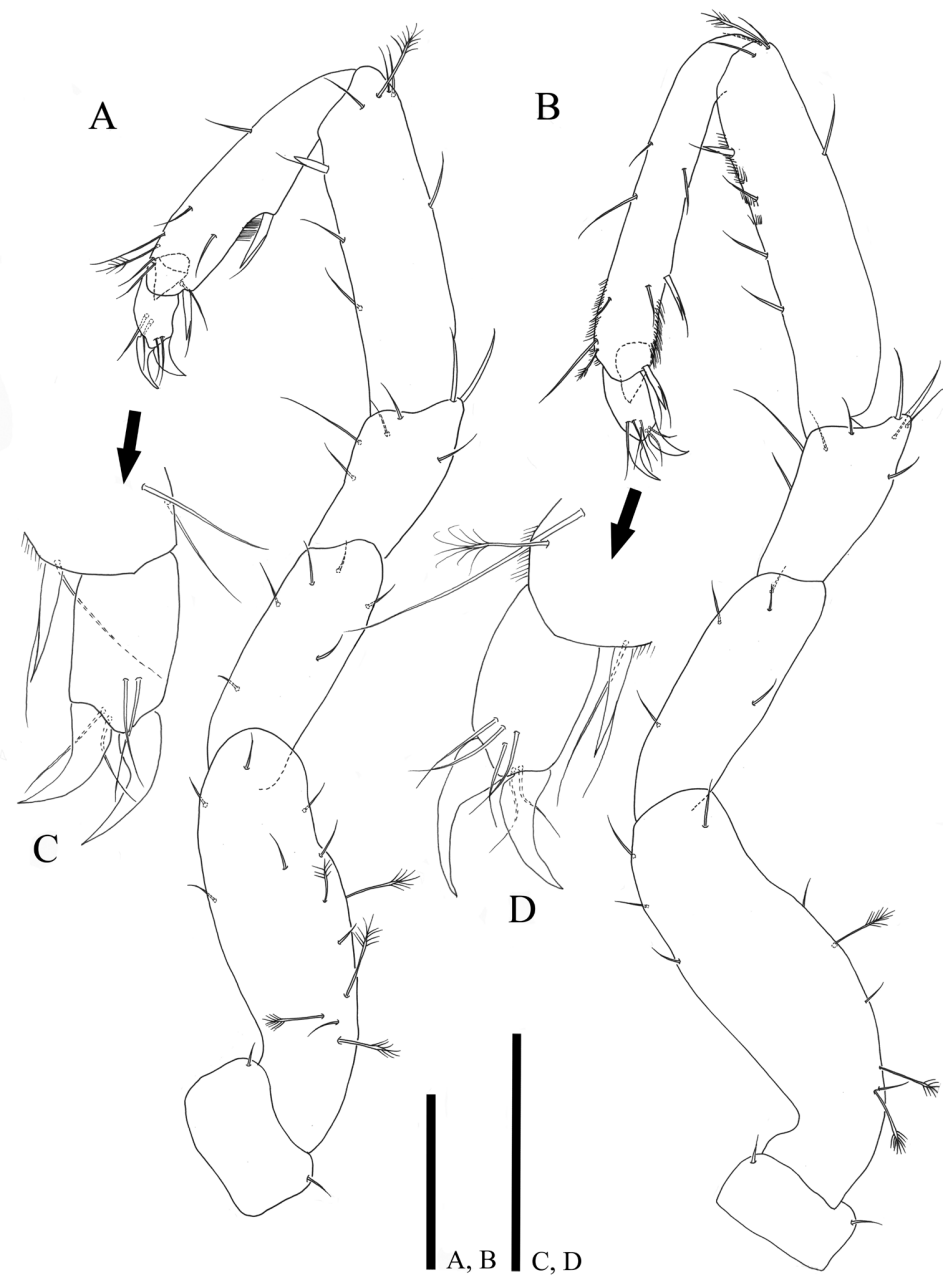
*Caecianiropsisgoseongensis* sp. n. holotype, male. **A** Pereopod 5, ventral, ×400 **B** pereopod 6, ventral, ×400, **C** propodus of left pereopod 5, ventral, ×1000 **D** propodus of pereopod 6, ventral, ×1000. Scale bar: 100 µm (**A, B**).

**Figure 7.**
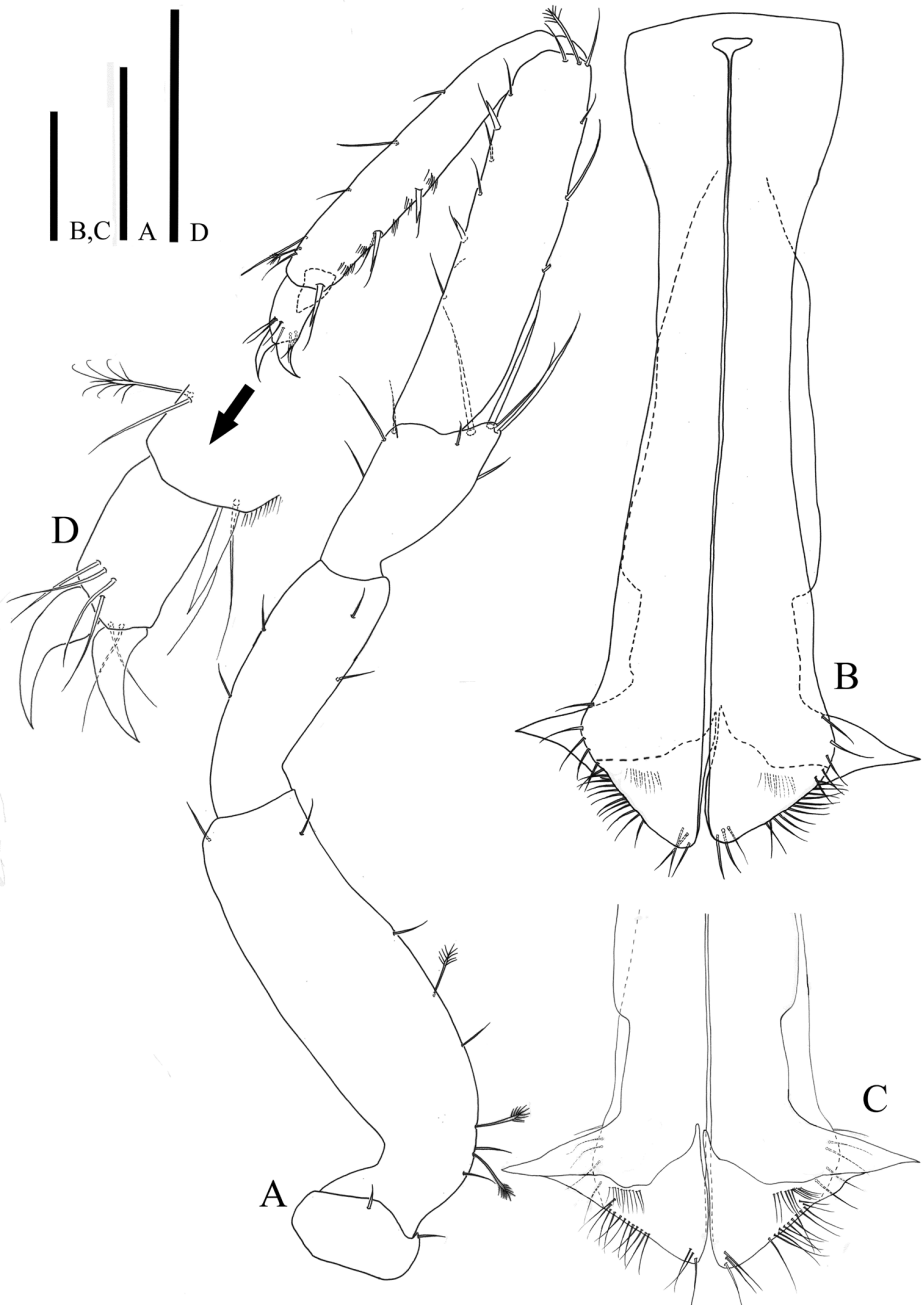
*Caecianiropsisgoseongensis* sp. n. holotype, male. **A** Pereopod 7, dorsal, ×400 **B** pleopod 1, ventral, ×400 **C** dorsal side of pleopod 1, ×400 **D** propodus of pereopod 7, ventral, ×1000. Scale bar: 100 µm.

*Posterior pereopods* (Figs 6A, B, 7A) inserted on pereon posterolaterally, relative length ratio in comparison to pereopod I, 1: 1.12: 1.27: 1.31; L/W ratio of articles: basis (3.1: 3.11: 3.08), ischium (2.86: 2.72: 2.72), merus (1.48: 1.67: 1.76), carpus (3.43: 4.17: 4.42) propodus (3.53: 6.69: 5.56), dactylus (1.45: 1.56: 1.45); coxae of pereopods V‒VII approximately twice as wide as those of anterior pereopods, clearly visible in lateral view with two simple setae; dactylus partly covered by articular plate projected from distal margin of propodus.

*Pereopod V* (Fig. 6A): basis dorsal margin with six simple and five broom setae, ventral margin with two setae; ischium with six simple setae; merus with seven setae of different length; carpus with six simple, one broom seta and one UB seta along margin; propodus with relatively bigger UB seta protruding medially, with seven simple setae, one broom seta, and one UB seta; dactylus (Fig. 6C) with two claws and four simple setae distally.

*Pereopod VI* (Fig. 6B): basis with seven simple three broom setae; ischium with five simple setae; merus with nine simple setae on distal margin; carpus with six simple, one broom, and one UB setae, spiny row along dorsal margin; propodus with seven simple setae, one broom seta, and two UB setae; dactylus (Fig. 6D) with two claws and six simple setae distally.

*Pereopod VII* (Fig. 7A): basis with six simple three broom setae, ischium with four setae; merus with nine simple setae on distal margin and three of them much elongate; carpus with nine simple, one broom seta, and two UB setae, spiny row along dorsal margin; propodus with six simple, one broom, and three UB setae; dactylus (Fig. 7D) with two claws and six simple setae.

*Pleopod I* (Fig. 7B): reaching posterior margin of pleotelson, consisting of two coalescent halves, elongate, 3.86 times longer than maximum wide (measured at widest section of proximal part); proximal part enlarged and becoming narrower until half of total length and subsequently broaden distally, 3.86 times longer than maximum wide (measured at widest section of proximal part); separated in half by medial stylet-guiding groove running from triangular opening on proximal part of medial groove, distolateral edge of hyaline lamella angular, projecting laterally; each distomedial lobe tapering distomedially, with ten simple setae distolaterally; distodorsal margin with 22 simple setae; 30 simple setae along distal apex, two distodorsal protrusions developed proximally (Fig. 7C).

**Figure 8.**
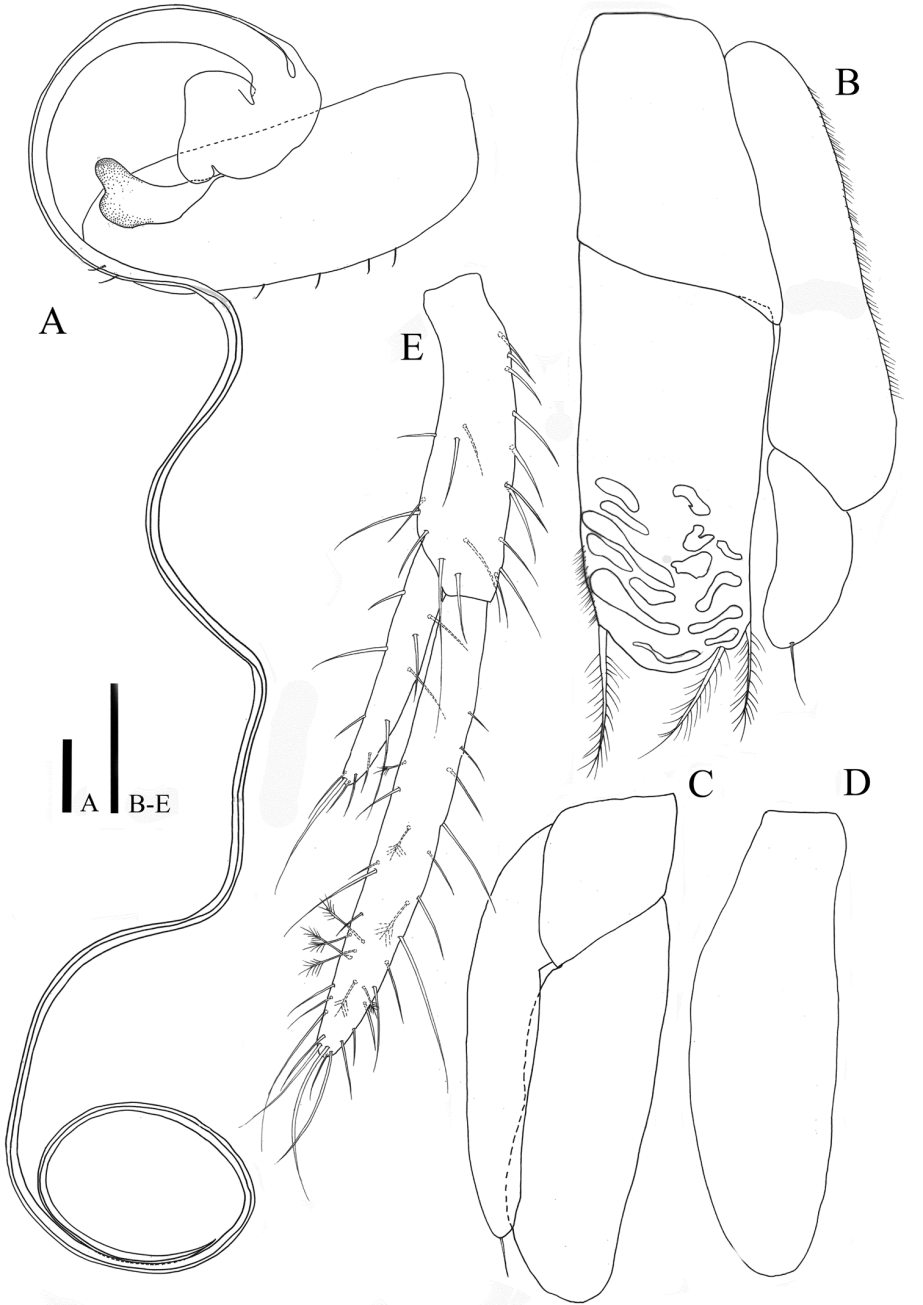
*Caecianiropsisgoseongensis* sp. n. holotype, male. **A** Pleopod 2, dorsal, ×400 **B** pleopod 3, dorsal, ×400 **C** pleopod 4, dorsal, ×400 **D** pleopod 5, dorsal, ×400 **E** uroopod, dorsal, ×400. Scale bar: 100 µm.

*Pleopod II* (Fig. 8A): sympod elongate, 2.7 times longer than wide; endopodal stylet, elongate, coiled in relaxed position, over 3 times longer than sympod, proximally robust but becoming narrower until distal tip, distal end of sperm duct consisting two rami (Fig. 10H) without ornamentation; exopod distal apex round, located below stylet, distomedially in sympod.

*Pleopod III* (Fig. 6C): sympod 1.61 times longer than wide; endopod length 0.67 times of sympod, 2.17 times longer than wide, suboval, with ornamentation like turtle shell shape, and with three plumose setae along distal margin; exopod two-segmented, basal segment 5.37 times longer than wide, with hairs along lateral margin, apical segment tapering distally, with one simple seta.

*Pleopod IV* (Fig. 7B): sympod, pentagonal, exopod 1.6 times longer than endopod, exopod tip not reaching to endopod tip; without ornamentation, exopod proximally curved, with distal seta.

*Pleopod V* (Fig. 7C): 3.16 times longer than wide, without rami and ornamentation, distal apex rounded.

*Uropods* (Fig. 7D): length 0.92 times of pleotelson; sympod robust, 3.5 times longer than wide, with 19 simple setae, most of setae on medial margin; endopod length 1.51 times of sympod with 24 simple and nine broom setae; exopod 0.5 times of endopod, with 12 simple setae.

*Penial papillae* (Fig. 10D, E): pointed distally, located ventrally on posteromedial margin of pereonite VII.

####### Sexual dimorphism of female.

**Figure 9.**
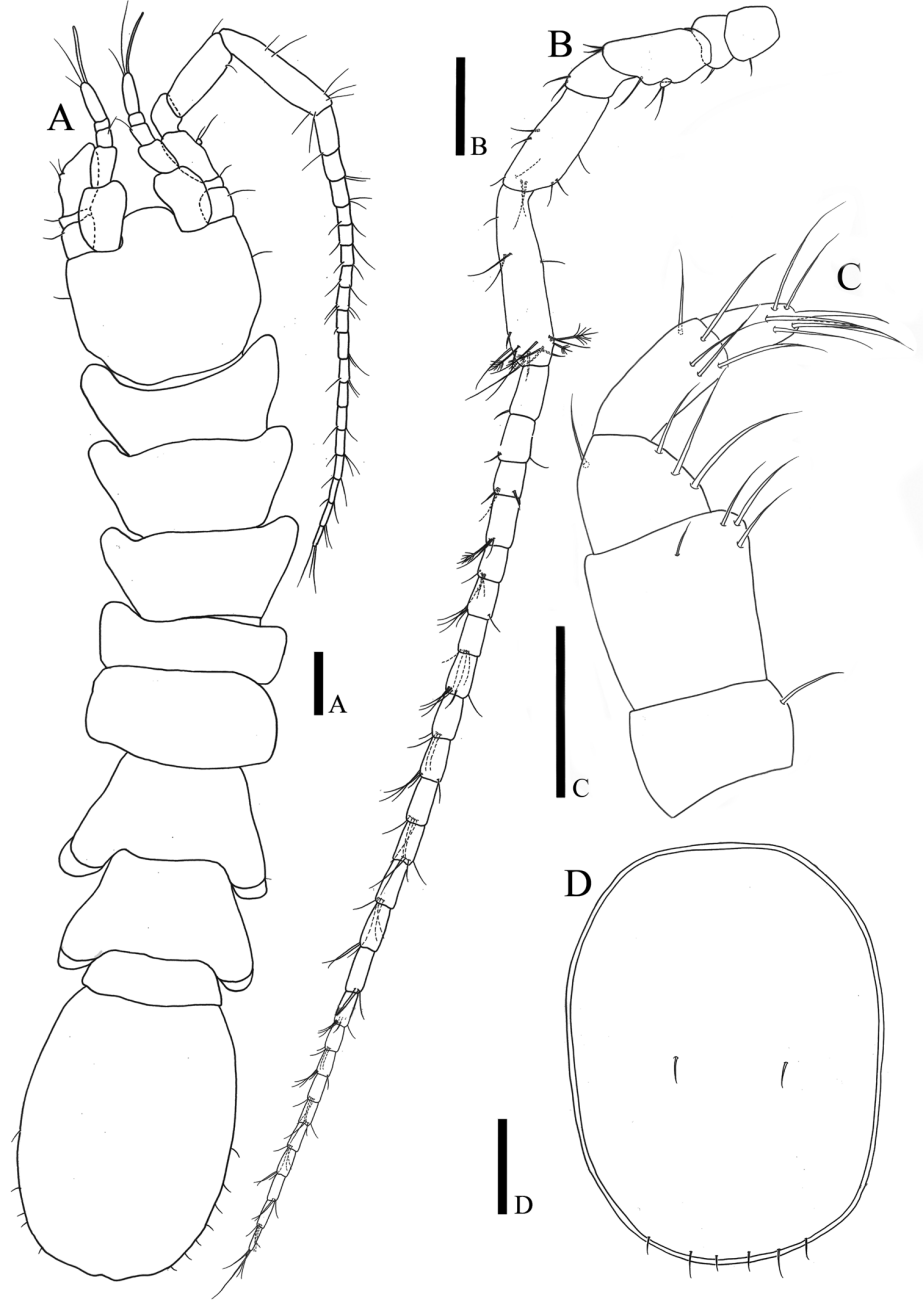
*Caecianiropsisgoseongensis* sp. n. allotype, ovigerous female. **A** Habitus, dorsal, ×200 **B** antenna, dorsal, ×400 **C** maxillipedal palp, ventral, ×1000 **D** operculum, dorsal, ×600. Scale bars: 100 µm (**A, B, D**), 50 µm (**C**).

*Body* (Fig. 9A): coxae of pleonites VI and VII visible in dorsal view.

*Antennula* (Fig. 9A): with five articles.

*Antenna* (Fig. 9B): with 25 flagellar articles.

*Maxillipedal palp* (Fig. 9C): article I with one seta distomedially, article II with four setae distomedially, article III with four setae, article IV with five setae, article V with seven setae distally.

*Female operculum* (Fig. 8D): 1.32 times longer than wide, two simple setae on dorsal surface, six setae along distal apex.

**Figure 10.**
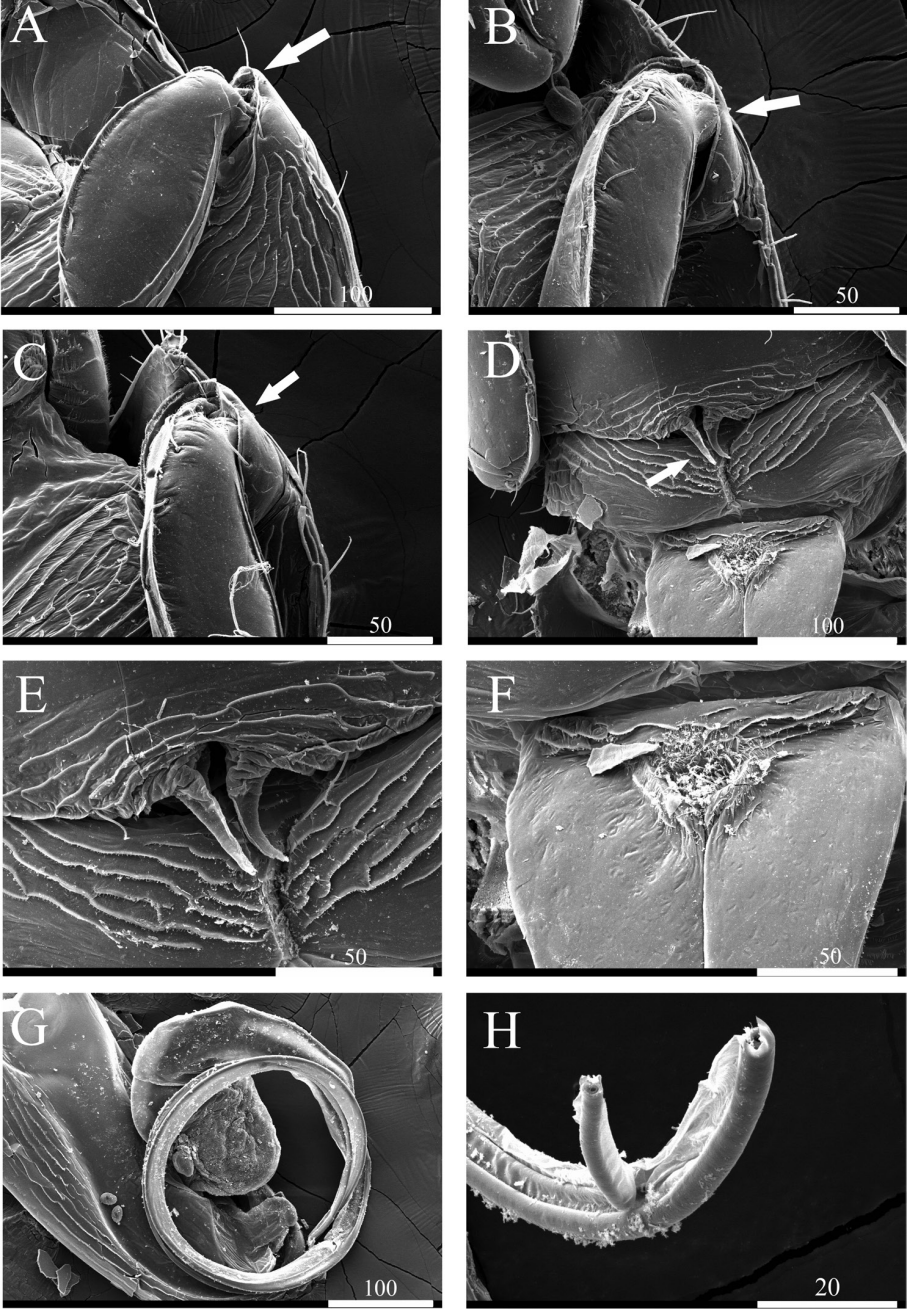
Scanning electron microscope images of *Caecianiropsisgoseongensis* sp. n. paratype 2, male. **A** Coxa of pereopod 1, ventral, ×450 **B** coxa of pereopod 2, ventral, ×600 **C** coxa of pereopod 3, ventral, ×600 **D** penial papillae, ventral, ×400 **E** penial papillae, ventral, ×900 **F** basal part of pleopod 1, ventral, ×800 **G** pleopod 2 with stylet, ventral, ×300 **H** distal tip of stylet, ventral, ×2000. Scale bars: 100 µm (**A, D, G**), 50 µm (**B, C, F**), 20 µm (**H**).

####### Etymology.

The new species is named after the type locality, Goseong, Gangwondo, Korea.

##### Key to the species of *Caecianiropsis* (males only)

The key is mainly based on the male characters.

**Table:** 

1	Rostrum approx. 1.6 times broader than article I of antennula; male pleopod I without distolateral angular projection	*** C. ectiformis ***
‒	Rostrum as wide as article I of antennula; male pleopod I with distolateral angular projection	**2**
2	Antennula with 7 articles	***C.goseongensis* sp. n.**
‒	Antennula with fewer than 7 articles	**3**
3	Endopod of uropod approx. 1.5 times longer than exopod	*** C. birsteini ***
‒	Endopod of uropod approx. 4 times longer than exopod	*** C. psammophila ***

##### 16S rRNA amplification

The final length of the trimmed sequence was comprised of 495 base pairs (GenBank accession numbers MH899013 and MH899014). BLAST (Altschul et al. 1990) of the GenBank database revealed that the obtained sequences were isopod in origin and not contaminated. *Microcharontanakai* Kim, Lee & Karanovic, 2017 (accession number KY498031.1, Lepidocharontidae) was the most similar sequence to *C.goseongensis* sp. n. resulting from Megablast optimization with 82% identity, 2e‒114 E‒value, 99% of query cover, 424 of total, and max score.

## Discussion

### Morphological comparison

*Caecianiropsisgoseongensis* sp. n. is similar to *C.psammophila* described from northern California in having the second mandibular palp with three serrate setae, truncate molar process, and also in the appearance of the male pleopod I and III. Their differences, however, include: the number of antennular articles (7 vs. 6); the ratio of maxillipedal epipodite (3.56 vs. 2.72), the length ratio of uropodal exopod to endopod (0.8 times of endopod vs. 0.24 times of endopod). *Caecianiropsisbirsteini* from Bering and Okhotsk Seas is easily distinguished from the new species by the five segmented antennula, nine apical setae on the maxillula, absence of refraction from the distal margin of male pleopod I, and by the relative length of uropodal exopod to endopod (69% vs. 49 %). Furthermore, differences in setal formula were observed in several appendages including antennula, antennal flagellum, pereopod I, uropodal rami, and the distal margin of pleopod I. The only southern species, *C.ectiformis*, has several distinct characteristics of which the most prominent are: the rostrum broader than antennule article I, the L/W ratio maxillipedal basis (1.4 vs. 3.1), the absence of the distolateral angular projection in the male pleopod I, and, the invisibility of uropodal sympod in dorsal view.

The non-ovigerous female of *C.goseongensis* sp. n. can be distinguished from the male primarily by its smaller body size. The other differences include five segmented antennulae, ratio of the antennular article V (3.27 vs. 2.34), antennula with fewer asthetascs (1 vs. 3), setal formula of the antennular article I and II, setal formula of the antennal article V and VI, and setal formula of all articles of the maxilliped palp. Although, Menzies and Pettit (1956) and Kussakin (1979) described the female operculum of both *C.psammophila* and *C.birsteini*, there was, however, no more information provided on the sexual dimorphic characters. Unlike all other species of the genus the operculum of *C.goseongensis* sp. n. bears several simple setae on the medial margin of the dorsal surface.

### Note on the habitat

The porosity and the volume of water permeating the interstitial space can be influenced by the particle size, which is one of the major factors characterizing the interstice (McLachlan and Turner 1994). Microorganisms inhabiting sediments are dependent on water inflow containing organic substance and minerals, essential for their life (Swedmark 1964). It has also been proven that the composition of many taxa in the sediment is closely related to the certain size or shape of particles (Dahl 1952; Jansson 1967; McLachlan 1996; Strayer et al. 1997; Defeo and Gómez 2005; De troch et al. 2006).

Menzies and Pettit (1956) noted that *C.psammophila* was collected from interstitial water on a coarse sand beach, while the other records of *Caecianiropsis*, including *C.goseongensis* sp. n., are from the shallow sub-littoral zone. Oh et al. (2007) carried out a granulometric analysis to infer the composition of sand particle in Naksan Beach (Gangwondo province, Korea). They found that the average particle size on the beach was much larger than that of the sub-littoral zone. Kim and Song (2012) also obtained similar granulometric results using sand samples from several beaches of Gangwondo province, where the type locality of *C.goseongensis* sp. n. is located. They collected the sand from five stations on each beach with 300 m intervals from the shoreline to the sub-littoral zone and measured the average diameter of particles. According to their results, the composition of sand particles and grain size tend to become smaller with distance, so that the sub-littoral zone has a finer substrate. Based on these results, *C.goseongensis* sp. n. is also distinguished from *C.psammophila* by the size of particles it lives in. This also may indicate that there is an interspecific preference for sand grain size in *Caecianiropsis*. However, data on the specific habitat type for the other *Caecianiropsis* is lacking.

## Supplementary Material

XML Treatment for Caecianiropsis

XML Treatment for Caecianiropsis goseongensis
